# Non-invasive biomarkers for sperm retrieval in non-obstructive patients: a comprehensive review

**DOI:** 10.3389/fendo.2024.1349000

**Published:** 2024-04-16

**Authors:** Laura Fontana, Silvia M. Sirchia, Chiara Pesenti, Giovanni Maria Colpi, Monica R. Miozzo

**Affiliations:** ^1^ Medical Genetics Unit, Aziende Socio Sanitarie Territoriali (ASST) Santi Paolo e Carlo, Milan, Italy; ^2^ Medical Genetics, Department of Health Sciences, Università degli Studi di Milano, Milan, Italy; ^3^ Next Fertility Procrea, International Center for Assisted Reproductive Technology, Lugano, Switzerland

**Keywords:** non-obstructive azoospermia, non-invasive biomarkers, infertility, reproductive medicine, TESE

## Abstract

Recent advancements in reproductive medicine have guided novel strategies for addressing male infertility, particularly in cases of non-obstructive azoospermia (NOA). Two prominent invasive interventions, namely testicular sperm extraction (TESE) and microdissection TESE (micro-TESE), have emerged as key techniques to retrieve gametes for assisted reproduction technologies (ART). Both heterogeneity and complexity of NOA pose a multifaceted challenge to clinicians, as the invasiveness of these procedures and their unpredictable success underscore the need for more precise guidance. Seminal plasma can be aptly regarded as a liquid biopsy of the male reproductive tract, encompassing secretions from the testes, epididymides, seminal vesicles, bulbourethral glands, and prostate. This fluid harbors a variety of cell-free nucleic acids, microvesicles, proteins, and metabolites intricately linked to gonadal activity. However, despite numerous investigations exploring potential biomarkers from seminal fluid, their widespread inclusion into the clinical practice remains limited. This could be partially due to the complex interplay of diverse clinical and genetic factors inherent to NOA that likely contributes to the absence of definitive biomarkers for residual spermatogenesis. It is conceivable that the integration of clinical data with biomarkers could increase the potential in predicting surgical procedure outcomes and their choice in NOA cases. This comprehensive review addresses the challenge of sperm retrieval in NOA through non-invasive biomarkers. Moreover, we delve into promising perspectives, elucidating innovative approaches grounded in multi-omics methodologies, including genomics, transcriptomics and proteomics. These cutting-edge techniques, combined with the clinical and genetics features of patients, could improve the use of biomarkers in personalized medical approaches, patient counseling, and the decision-making continuum. Finally, Artificial intelligence (AI) holds significant potential in the realm of combining biomarkers and clinical data, also in the context of identifying non-invasive biomarkers for sperm retrieval.

## 1 Introduction

Azoospermia is primarily classified into Obstructive Azoospermia (OA) and Non-Obstructive Azoospermia (NOA). A multitude of causes and risk factors contribute to male infertility, which can be stratified as congenital, acquired, or idiopathic (1). OA is characterized by the absence of sperm in the ejaculate due to a physical blockage or obstruction within the male reproductive tract. This obstruction can occur at various anatomical sites, including epididymis, vas deferens, or ejaculatory ducts. While spermatogenesis is usually normal or only mildly impaired in OA, spermatozoa can still be retrieved from the testis or epididymis for assisted reproductive technologies (ART). The causes of OA range from congenital anomalies, sometimes related with genetic diseases like cystic fibrosis, to acquired conditions, such as infections, surgical outcomes, or trauma (2–4). In contrast, NOA involves a complete absence of sperms in the ejaculate due to impaired spermatogenesis. It affects about 1% of all men and 10% of those with infertility (5). The etiology of NOA is heterogeneous, ranging from genetic anomalies and hormonal imbalances to exposure to toxic agents or varicocele (6). Approximately 30–50% of male infertility cases are idiopathic, with environmental exposure to toxic chemicals and lifestyle habits hypothesized as contributing factors (7).

While NOA is defined by the absence of spermatozoa in the ejaculate, the presence of focal areas of spermatogenesis within the testes cannot be excluded, and sperm retrieval is possible using testicular surgical procedures when active spermatogenic foci are found. Advances in reproductive medicine have led to reliable approaches, like testicular sperm extraction (TESE) and microdissection TESE (m-TESE) becoming key interventions in NOA for ART (8). However, these procedures are not uniformly successful, with outcomes heavily depending on the etiology of the condition, including genetic factors and hormonal profile variations (9). Despite routine evaluation to define the etiology in NOA patients, predicting sperm retrieval outcomes remains challenging, underscoring NOA complexity for clinicians.

Recently, there has been growing interest in non-invasive biomarkers to predict sperm presence in NOA patients’ testes (10). These approaches are promising as they offer safer, less invasive alternatives to identify patients more likely to benefit from successful sperm retrieval, thus optimizing ART procedures and potentially reducing unnecessary surgeries. However, the clinical application of molecular biomarkers for predictive purposes in NOA is still limited, mainly due to the heterogeneity of the condition. Incorporating clinical data with robust biomarkers to predict the success of surgical procedures and guide their selection could significantly impact the clinical management of NOA.

Once diagnosed, various clinical, biochemical, and molecular markers associated with NOA are crucial for defining the etiology. These include:

-Clinical parameters: the testicular volume and ultrasound characteristics may correlate with the likelihood of successful sperm retrieval (11). Additionally, the histological analysis of testicular biopsies is important for determining the stage of spermatogenic damage.
-Hormonal profile: the hypothalamic-pituitary-gonadal (HPG) axis plays a central role in regulating spermatogenesis. The gonadotropin-releasing hormone (GnRH) from the hypothalamus stimulates the pituitary to release the luteinizing hormone (LH) and follicle-stimulating hormone (FSH), which in turn stimulate Leydig and Sertoli cells in the testes. Elevated FSH levels, along with low inhibin B, can indicate spermatogenic failure, although they are not specific indicators of the arrest stage. LH and testosterone levels also provide insights into the hormonal environment affecting NOA (12).
-Genetic etiology: identifying karyotype anomalies, Y-chromosome microdeletions, or genetic syndromes associated with NOA aids in prognosis and management (7). Recent advancements in Whole-Exome Sequencing (WES) have revealed additional genetic variants that can be associated with spermatogenic failure and therefore predict the effectiveness of surgical procedures (13, 14).
-Molecular profiling and epigenetic determinants: the pursuit of molecular non-invasive biomarkers, capable of predicting the presence, quality, and quantity of spermatozoa within the testicular microenvironment, has gained significant attention. The integration of genomics, transcriptomic, proteomics, and metabolomics is revolutionizing our understanding of the molecular complexities of spermatogenesis (15). Seminal plasma, urine, and blood are key biological sources for exploring molecular and proteomic signatures associated with spermatogenesis. Seminal plasma, in particular, is a promising liquid biopsy medium in NOA patients, containing cell-free nucleic acids, microvesicles, proteins, and metabolites linked to gonadal activity (16). In particular, circulating microRNAs have emerged as promising biomarkers due to their stability in body fluids and their role in regulating genes relevant to spermatogenesis (17).

Spermatogenesis involves extensive biochemical and morphological changes in round spermatids, including chromatin condensation, acrosome formation, and flagellum assembly, culminating in the production of mature spermatozoa. During gametogenesis, chromatin undergoes significant modifications, such as histone alterations and the transition to protamines, to ensure proper sperm development, DNA repair, and chromosomal segregation.

The transition from spermatogonia to mature spermatozoa is governed by a tightly regulated sequence of genetic and epigenetic modifications, essential for the proper development and function of sperm cells. Genetic and epigenetic errors during spermatogenesis within the testicular environment can cause sperm cell production failure at various specialized steps. Understanding these specific alterations is essential for deciphering the mechanisms underlying male infertility.

Therefore, stratifying NOA patients should be based on the specific step of the gametogenesis arrest and the role of involved genes and their regulatory mechanisms. The histological analysis of testicular biopsy is the gold standard for determining the stage of spermatogenic damage, diagnosing conditions associated with NOA, and predicting sperm retrieval success (18, 19).

The Sertoli Cell-Only Syndrome (SCO) is defined when only Sertoli cells are present in the seminiferous tubules, with no germ cells found. This indicates a very early arrest, where spermatogonia are either absent or not developing properly.

Maturation arrest is diagnosed when germ cells are present but do not progress to the stages of gametogenesis. The arrest can occur at the level of spermatogonia, primary spermatocytes, or secondary spermatocytes.

Hypospermatogenesis is characterized by a reduced number of germ cells, which may progress through all stages of spermatogenesis but in significantly reduced numbers, leading to occasional or no spermatozoa in the ejaculate.

Given the clinical and etiological diversity of NOA (1), an integrated approach encompassing clinical evaluations, genetic testing, biochemical assays, and other molecular profiling (e.g. transcriptomic and metabolomic analyses) could provide clinicians with a more comprehensive understanding than single-marker reliance. This personalized medical strategy has the potential to enhance the precision of sperm recovery prediction, thereby improving clinical decision-making. Moreover, Artificial intelligence (AI) holds significant promise in combining biomarkers and clinical data, especially in identifying non-invasive biomarkers for sperm retrieval (20).

This review aims to critically evaluate the genetics of NOA, the current knowledge of molecular non-invasive biomarkers in NOA patients, and their applicability to clinical practice. Special attention is paid to unique genetic profiles associated with spermatogenesis failure, which might be considered as signatures of NOA in a subset of cases.

## 2 Biological sample for the analysis of non-invasive biomarker in NOA

In recent studies, significant efforts have been made in identifying potential non-invasive biomarkers for NOA. Several biological samples can be used to identify potential non-invasive biomarkers for NOA, which include:

### 2.1 Seminal plasma

This is a primary sample used for the study of NOA. It can provide valuable information through the analysis of sperm DNA, RNA (including microRNAs and other non-coding RNAs), cell free DNA (cfDNA), proteins and metabolic profiles. Seminal plasma analysis can reveal insights into sperm function, spermatogenesis, and the underlying causes of NOA (21).

### 2.2 Urine

Urine can be used to detect biomarkers associated with reproductive health. It can contain DNA, RNA, hormones, and metabolites that reflect the physiological state of the reproductive system (22).

### 2.3 Peripheral blood

Peripheral blood is a common non-invasive sample source for biomarker research. Blood tests can measure hormone levels, genetic material, and other circulating molecules that could be indicative of NOA or related conditions (23).

### 2.4 Saliva

Although less commonly used than other samples, saliva can contain hormones and other biomolecules that might reflect reproductive health status, including conditions like NOA (24).

### 2.5 Epididymal fluid

In some cases fluid from the epididymis can be collected and analyzed, particularly when looking at sperm maturation processes.

Please separate this paragraph each of these samples can offer unique information about the reproductive system and all these biological samples may be useful in identifying biomarkers for the diagnosis and treatment of NOA. The choice of sample often depends on the specific biomarker of interest, the ease and feasibility of collection, and the invasiveness of the procedure.

## 3 Potential non-invasive biomarkers in NOA

Non-invasive biomarkers in NOA are classified based on their biological nature and the type of information they provide. A summary of non-invasive biomarkers in NOA are reported in **Table 1**.

**Table 1:** Summary of non-invasive biomarkers in NOA.

Marker	Biological sample	Methodological approach	Limitations
Chromosomal abnormalities	Blood	Karyotyping	Routinely performed low resolution
Gene variants	Blood, saliva	NGS	Performed only in selected cases based on clinical findings
cfDNA/cfRNA levels	Seminal plasma	NGS, PCR-based methods	Not specific markers; levels could be influenced by various factors unrelated to the condition being tested.
Transcriptomics	Seminal plasma, testicular biopsy	NGS, RT-PCR	Moderate specificity, challenges in achieving accurate and sensitive detection
Proteomics	Seminal plasma, testicular biopsy	Liquid chromatography-tandem mass spectrometry (LC-MS/MS), immunodiagnostic test	Highly specific only for some proteins, high costs

### 3.1 Genetic defects

Collectively, genetic factors are believed to underlie approximately 30% of NOA cases (25). Karyotype anomalies, and Y chromosome microdeletions associated with NOA are summarized in **Table 2**. Among them Klinefelter syndrome (47,XXY also in a mosaic condition) represents the most frequent cytogenetic abnormality associated with NOA.

**Table 2:** Karyotype anomalies and Y chromosome microdeletions associated with NOA.

Chromosomal alterations	Reproductive consequences	Frequency in NOA	Sperm retrieval	Offspring outcome	Reference
Klinefelter syndrome (pure and mosaic conditions)	Deterioration of testicular tissue	10-12%	34-44%	Normal karyotype in offspring	(26)
Chromosomal translocations	Meiosis arrest for impaired homologues chromosomes pairing	1-2%	Variable	High risk of chromosomal unbalancies, UPD (uniparental disomies)	(27)
Robertsonian translocations		0.9-3.4%			
Reciprocal translocations		0.1-0.3%			
DSD, XX (SRY+)	Azoospermia	Very rare	0%		(28)
Structural abnormality of Y chromosome	Depends on the AZF specific deletion	Rare	Variable	Inheritance of the marker Y in males	(29)
AZF deletions	Depends on the deleted region	7-10%		AZF deletion in males	(27, 30, 31)
AZFa	SCOS		0%		
AZFb	Meiotic arrest		0%		
AZFc	Variable		50-60%		

Routine clinical assessment of NOA patients typically includes karyotyping, evaluation of microdeletions in the Y chromosome AZF region, and, in selected cases, gene sequencing to reveal possible monogenic conditions. When sperm retrieval is possible, genetic alterations identification in NOA patients is crucial for understanding potential impact on offspring, therefore it is important to discuss the chance of genetic defect transmission during genetic counseling, along with options for pre-implantation or prenatal diagnosis. For example, in case of balanced chromosomal translocations, the production of unbalanced gametes, resulting in infertility, offspring with severe phenotypes, or miscarriages, should be deeply discussed with the couple before ART procedures.

Another chromosomal cause of NOA relates in structural alterations of Y chromosome, which can be cytogenetically visible (e.g. translocation involving Y; deletions or duplications of the entire Yq arm) or submicroscopic, as in case of deletions affecting the AZF regions on the Yq. The AZF is classically divided in three regions (AZFa, AZFb, AZFc), harboring genes essential for spermatogenesis. Identifying specific AZF deletions helps to predict the success rate of surgical sperm retrieval (32) (**Table 2**). A proposed mechanism for the different outcomes associated with deletions of AZFa, AZFb, AZFc focuses on the importance of the *DAZ* gene, characterized by several copy number variants (CNVs) and located in the AZFc region (DAZ1, DAZ2, DAZ3, and DAZ4). This genomic structure of AZF regions, enriched in CNVs, predisposes them to microdeletions (33). The complete deletion of the AZF regions is also present in males with a female karyotype, a case belonging to the Disorders of Sex Development (DSD), XX subgroup. The male phenotype is related to the presence of the *SRY* gene on one X homologue, due to an incorrect crossing-over in the father of the proband involving the pseudoautosomal regions on the p arm of X and Y chromosomes (29).

Regarding gene variants in NOA, the introduction of high-throughput sequencing methods has significantly increased the identification of candidate genes associated with genetic disorders, including male infertility.

Pathogenic variants in genes associated with infertility have been reported in both isolated and syndromic NOA cases (6).

A recent systematic review of the validated monogenic causes of human male infertility is reported by Houston et al. (34). Of the hundreds of genes expressed in testis, the most notable group belongs to the “*TEX* genes” (i.e. male-germ-cell-specific genes). This term, coined by Wang et al. (35), refers to the about 60 human genes distributed throughout the genome, playing roles in spermatogenesis and fertilization (36–39).

In a recent study using WES to improve the diagnosis of men with NOA (14), the authors employed a straightforward strategy based on a set of candidate genes to aid in interpreting WES data. This study identified causative mutations in genes with predicted or well-established impact on spermatogenesis in the 23% of NOA subjects, thus doubling the detection rate of NOA patients with a genetic defect. In addition, another study (40) evaluating 35 patients with spermatogenic failure reported causative mutations in 20 different genes in the 5.4% of analyzed subjects.

These findings highlight the potential of the genetic diagnosis, when combined with other clinical data, to provide strong guidance regarding the suitability of TESE. They support the use of NGS in assisting clinicians in offering more precise and relevant advice to patients, potentially influencing treatment decisions. Among the genes mutated in NOA reported by Kherraf et al. (14), and others (41), several (e.g. *C14orf*39, *HFM1*, *MCM8*, *MEIOB*, *MSH4*, *DMC142*, *STAG3, KASH5*) are also associated with female infertility, underscoring common mechanisms in male and female meiosis. A comparative evaluation of recent studies (14, 34, 40, 42) has reported pathogenic or likely pathogenic variants in genes like *M1AP*, *TDRD9*, *TEX11, and KASH5* in NOA patients. Some of the more relevant genes found mutated in NOA patients are summarized in **Table 3**. The list includes:

**Table 3:** Most relevant genes mutated in NOA patients.

Gene	OMIM	Protein	Inheritance	Function	Phenotype	Reference
*M1AP*	619098	Meiosis 1-associated protein	Recessive	Likely to function in meiosis progression. In mouse plays a role in gametogenesis in both sexes	Meiotic arrest and severely impaired spermatogenesis	(43)
*TDRD9*	617963	Tudor Domain-Containing Protein 9	Recessive	Transposon silencing in spermatogenesis maintaining DNA stability	Maturation arrest	(44)
*TEX11*	300311	Testis-expressed gene 11	X-linked	Meiotic progression in late spermatogenesis	Meiotic arrest and severely impaired spermatogenesis	(45)
*KASH5*	618125	Coiled-Coil Domain Containing 155	Recessive	Component of the LINC (LInker of Nucleoskeleton and Cytoskeleton) complex, involved in the connection between the nuclear lamina and the cytoskeleton	Prepachytene meiotic arrest	(46)
*MSH4*	602105	MutS Protein Homolog 4	Recessive	Meiosis-specific protein required for reciprocal recombination and homologous chromosomes segregation at meiosis I	Meiotic arrest	(47)

The *M1AP* gene (Meiosis 1-Associated Protein, OMIM: 619098) that is crucial for meiosis, and its dysfunction can lead to primary testicular failure, a major contributor to NOA. Variants in *M1AP* have been associated with cryptozoospermia and oligozoospermia in three different studies (43, 48, 49).

*TDRD9* (Tudor Domain-Containing Protein 9, OMIM: 617963), is essential for transposon silencing in spermatogenesis. Biallelic variants in *TDRD9* have been linked to maturation arrest in azoospermic men (44).

The X-linked *TEX11* (Testis-Expressed gene 11, OMIM: 300311) gene, expressed in late-pachytene spermatids, is absent in Sertoli and interstitial cells, indicating its specific role in germ cells. Specifically, *TEX11* is involved in the association of homologous chromosomes and meiotic recombination. Hemizygous mutations in *TEX11* were found in 2.4% of azoospermic men (45).

The *TEX15* (Testis Expressed 15, OMIM: 605795) gene encodes a protein that is required for DNA double-strand break repair, chromosome synapsis, and meiotic recombination in spermatocytes. Males caring homozygous variants in *TEX15* show spermatogenesis defects that result in severe oligozoospermia or azoospermia (50). However, *TEX15* mutations are very rare in NOA.

Rare recessive forms of NOA involve *KASH5* (Coiled-Coil Domain Containing 155, OMIM: 618125), which protein links the nucleoskeleton and cytoskeleton by interacting with the inner nuclear membrane protein SUN1 and recruiting the cytoplasmic protein dynein (LINC complex). The nucleocytoplasmic interactions established by the LINC complex is required for telomere attachment to the nuclear envelope in the prophase of meiosis and for rapid telomere prophase movements in both male and female gametogenesis (51). Pathogenic variants in *KASH5* have been reported in consanguineous families. Male infertility associated with *KASH5* mutations is due to prepachytene meiotic arrest (46).

The *MSH4* gene (MutS Protein Homolog 4, OMIM: 602105) encodes for a meiosis-specific protein that is involved in DNA mismatch correction and is required for reciprocal recombination and proper segregation of homologous chromosomes at meiosis I. Biallelic variants are associated with premature ovarian failure and spermatogenic failure. Limited NOA cases have been reported to carry *MSH4* variants and were characterized by no round spermatids or spermatozoa in the seminiferous tubules (47).

In addition to genes involved in isolated NOA, syndromic forms have been also recognized. Among them: dynein genes known for their roles in ciliary movement, have been associated with syndromic forms of azoospermia, including Primary Ciliary Dyskinesia and Kartagener Syndrome (OMIM: 244400). These conditions feature additional symptoms beyond reproductive issues, such as respiratory system abnormalities. Dynein mutations disrupt axonemal structure, essential for sperm motility, highlighting a syndromic link to azoospermia (52).

The Wilms Tumor 1 (*WT1*) gene, critical for kidney and gonadal development, has been linked to syndromic NOA. *WT1* mutations can lead to disorders such as Denys-Drash syndrome (OMIM: 194080) and Frasier syndrome (OMIM: 136680), which include genital tract malformations and predisposition to Wilms tumor, alongside NOA (53).

Kallmann syndrome (OMIM: 308700) is a developmental genetic disorder characterized by the association of congenital hypogonadotropic hypogonadism (CHH) due to GnRH deficiency, and anosmia or hyposmia (with hypoplasia or aplasia of the olfactory bulbs).

Fanconi anemia (OMIM: 227650) can lead to reproductive issues, including NOA as part of its broad spectrum of clinical manifestations. This connection is primarily due to the role of the Fanconi anemia pathway in DNA repair, critical for healthy spermatogenesis. Krausz et al. (54), identified a high-risk subgroup of infertile men with NOA for undiagnosed Fanconi anemia, highlighting the importance of assessing FA-related genes mutations in selected NOA patients.

The diverse range of loci identified in various studies underscores the complex genetics of NOA, with these genes representing significant contributors to the condition, although in limited cases. Their identification and study offer insights into the molecular mechanisms of NOA and pave the way for potential therapeutic interventions and stratification of patients on the bases of possible genetic causes. Understanding the genetic alterations underpinning NOA has also broader implications in the field of reproductive medicine; the identification of genetic defects shared between male and female infertility suggests a more interconnected approach to understanding and treating reproductive disorders.

In conclusion, an extensive genetic evaluation of NOA subjects should be incorporated into the clinical assessment of patients not only in selected cases (**Table 1**), as it offers more chances for understanding the etiology of infertility, which can directly influence management and treatment strategies. The integration of karyotyping, AZF microdeletion analysis, and targeted gene panels assays should be considered the baseline for genetic assessment. In addition, with the advent of high-throughput sequencing technologies, the diagnostic yield is improving in NOA, enabling the identification of novel genetic factors contributing to NOA.

The genetic assessment can provide valuable insights into the likelihood of successful sperm retrieval by TESE, despite the complex and not fully elucidated relationship between mutations in genes associated with NOA and their specific histological manifestations.

In addition, genetic counseling becomes paramount in cases in which genetic abnormalities are detected. Patients and their partners should be informed of the implications for their offspring, including the risk of transmitting genetic abnormalities and the available options for pre-implantation genetic diagnosis (PGD) or prenatal testing.

Considering genes with strong evidence as well as those found vary rarely affected in NOA males, it might be more efficient the use of WES to identify pathogenic or likely pathogenic variants in NOA. This approach allows for the comprehensive analysis of genetic variants across all coding regions of the genome. Subsequently, virtual selection of genes known or suspected to be involved in NOA can further refine the search for causal mutations, streamlining the diagnostic process and enhancing our understanding of the genetic basis of NOA. This strategy optimizes the use of genetic information, facilitating the identification of potential targets for therapeutic intervention expanding the knowledge on the genetic basis of NOA. Furthermore, mutational analysis should be preceded by counseling, to collect the clinical family history to facilitate understanding of inheritance patterns in each family.

### 3.2 Cell free nucleic acids

Various studies have shown significantly higher mean cfDNA levels in seminal plasma from patients with sperm abnormalities compared to controls. The levels of cfDNA are particularly elevated in men with azoospermia, including NOA, and teratozoospermia, suggesting a strong association between cfDNA levels and sperm abnormalities. Two primary hypotheses for elevated cfDNA levels in NOA are: i) abortive spermatogenesis: in NOA, germinal stem cells may begin differentiation but are halted by checkpoints, leading to apoptosis and increased cfDNA levels; ii) infections and inflammatory phenomena: inflammation, whether specific or nonspecific, can cause cell lysis, releasing nucleic acids into the seminal plasma.

The majority of cfDNA in seminal plasma is believed to derived from apoptotic and necrotic cells within the male reproductive tract (55, 56). During apoptosis, cells undergo programmed cell death, leading to DNA fragmentation. Similarly, necrosis resulting from cellular injury can release DNA into the extracellular environment (57). Additionally, during spermatogenesis, germ cells undergo extensive remodeling, and DNA that is not incorporated into spermatozoa can be shed into the seminal fluid (58). Cells from the epididymis and prostate, which significantly contribute to seminal fluid, may release DNA during their physiological turnover or in response to inflammation or pathology. Moreover, cfDNA can be directly released from spermatozoa, particularly from those that are damaged or undergo cell death (16).

​The concentration of cfDNA in seminal plasma may serve as a predictive biomarker for the success of sperm retrieval in NOA. Elevated levels of cfDNA can, indeed, indicate abortive spermatogenesis or infections​ (59, 60).

Regarding cell free RNA (cfRNA), a significant portion of cfRNAs in seminal plasma is packaged within extracellular vesicles such as exosomes. These vesicles are secreted by various cells in the reproductive tract, including prostate epithelial cells, seminal vesicles and Sertoli cells (61).

RNAs are also released from cells at different stages of spermatogenesis. As these cells progress through various stages of maturation, they may shed RNAs into the seminal plasma. Like cfDNA, apoptosis of germ cells and somatic cells within the testes can lead to the release of RNAs into seminal plasma (62). In addition, prostate and seminal vesicles can release RNA molecules in the seminal plasma, thus reflecting the physiological and pathological state of these tissues (63).

Limitations: despite the growing interest in cell-free nucleic acid as non-invasive biomarkers for the diagnosis and prognosis of disease in various clinical contexts, including NOA, its clinical use in standard practice is limited for several reasons:

-Validation and standardization: there is a lack of standardization in the methods of collection, preservation, extraction, and analysis of cfDNA. Establishing uniform protocols is crucial to ensure the reliability and comparability of results.
-Sensitivity and specificity: the sensitivity and specificity of cfDNA and cfRNA as biomarkers need to be accurately determined. Although preliminary studies show promising results, further research is required to confirm the effectiveness of cfDNA in the diagnosing and prognosing of NOA.
-Underlying biological mechanisms: the mechanisms through which cfDNA levels are altered in NOA are not fully understood. A better understanding of these mechanisms could improve the correlation with specific clinical conditions.
-Variation in cfDNA levels: cfDNA levels can be influenced by a variety of factors, including other pathological conditions, inflammation, and even physical stress. These variations can make it challenging to interpret results specific to NOA without a clear understanding of the patient’s overall clinical context (**Table 1**).
-Cost and accessibility: the techniques for the analysis of cell-free nucleic acid are expensive and require specialized equipment not available in all clinical laboratories.

Although data on cfDNA are more extensive compared with those on cfRNA, additional clinical studies are needed to confirm the utility of cfDNA in various contexts of male infertility, to validate specific biomarkers, and to integrate cfDNA results with other diagnostic methods. It is conceivable that the search on cfDNA and cfRNA markers in seminal plasma might not yield consistent indicators due to the heterogeneous nature of NOA.

More comprehensive studies are crucial to establish the clinical utility of cfDNA and cfRNA as biomarkers in NOA. However, these studies should be meticulously designed considering the patient stratification on clinical and genetic background. This approach acknowledges that cfDNA and cfRNA may serve as specific indicators for different impediments in the spermatogenesis process, reflecting the unique pathophysiological conditions of each NOA case.

In conclusion, while cfDNA has significant potential as a diagnostic and prognostic tool in NOA, its practical application requires further research and development in the field of reproductive medicine. Understanding the origins of cfDNA and cfRNA in seminal plasma is crucial for interpreting their levels and modifications in different pathological or physiological conditions.

### 3.3 Transcriptomics

Many genes are expressed during meiosis, and their transcription profile was explored in NOA patients as biomarkers. They can be classified in: i) microRNAs (miRNAs), small non-coding RNAs that regulate gene expression, found in seminal plasma, urine, and blood; ii) messenger RNAs (mRNAs), reflective of gene expression patterns relevant to spermatogenesis; iii) circular RNAs (circRNAs), covalently closed RNA molecules, with roles in gene regulation; and iv) long non-coding RNAs (lncRNAs), involved in chromatin remodeling, transcriptional and post-transcriptional regulation.

Expression analyses in NOA are significantly improving our understanding on the molecular mechanisms underlying this condition, mainly by the evaluation of testes biopsies, which results can be tested and translated in semen plasma. In the past years, studies on single potential biomarkers have been performed, now overtaken by omics technologies. One example was the evaluation of *ESX1* gene transcript in NOA (64, 65).

The development of single-cell RNA sequencing has been, indeed, pivotal in this regard, enabling the investigation of individual cell populations in the testis at high resolution. Such studies have provided a deeper insight into the pathogenesis of NOA and identified key genes and transcription factors involved in this process (63).

The Transcriptional Regulatory Networks (TRNs) in single-cell RNA-Seq data sets, has identified specific transcription factors in Leydig cells and testicular macrophages of iNOA patients, such as *LHX9*, *KLF8, KLF4, ARID5B*, and *RXRG* in iNOA Leydig cells, and *POU2F2, SPIB, IRF5, CEBPA, ELK4, and KLF6* in iNOA testicular macrophages. Interestingly, about 256 differentially expressed genes (DEGs), including the upregulation of *TEX38, FAM71F, PRR30, FAM166A, LYZL6, TPPP2, ARMC12*, and *SPACA4*, have been identified in NOA. These genes are involved in crucial biological processes like spermatogenesis, cell differentiation, flagellated sperm motility, and spermatid development. These findings underscore the complexity of NOA and the diverse molecular pathways involved. The identified transcription profile and their regulatory mechanisms add knowledge in NOA pathophysiology and highlight potential areas for targeted interventions.

Translating these findings into the identification of non-invasive biomarkers for predicting the success of sperm retrieval in patients with NOA involves several steps: i) the identification of specific biomarkers in liquid biopsy, mainly in seminal fluid, since most studies have identified specific changes in the Sertoli cells of NOA patients. This might involve the detection of specific mRNAs or miRNAs that reflect the changes observed in testicular tissues. In addition, further studies are essential for their validation to verify their specificity and sensitivity in predicting sperm retrieval success across different patient groups; ii) the integration with other clinical findings: biomarkers derived from these studies should be considered alongside existing clinical indicators, such as hormone levels, genetic analysis, and physical examination, to provide a comprehensive and personalized assessment of sperm retrieval potential; iii) the technological development and accessibility: the implementation of non-invasive tests requires advanced technologies for the accurate and sensitive detection of biomarkers at very low levels. The accessibility and feasibility of such tests are crucial for their widespread clinical application.

In summary, while these results offer promising perspectives, their translation into non-invasive diagnostic tools for predicting sperm retrieval success requires additional research, technological development, and clinical validation.

Recently, a comprehensive analysis of the potential use of seminal plasma for biomarker detection in NOA patients to predict sperm retrieval was carried out by Li et al. (17). The authors evaluated about 80 studies ranging in quality from medium to high. The biomarkers examined were diverse, including RNAs (miRNAs, lncRNAs, circRNAs, tsRNAs). Within the vast array of RNA found in seminal plasma, specific types have been highlighted for their roles in cellular processes that may mirror the status of spermatogenesis. These include miRNAs, lncRNAs, circRNAs, and tRNA-derived small RNAs (tsRNAs). Several miRNAs are implicated in the development of germ cells and the process of spermatogenesis. Their stability in seminal plasma, due to their packaging in microvesicular bodies or protein complexes, makes them promising non-invasive markers for spermatogenesis. The study has shown a different expression of specific miRNAs in NOA patients with both positive and negative TESE outcomes, suggesting their potential in predicting spermatozoa retrieval, though further validation is needed.

LncRNAs are involved in various regulatory functions within the cell and have been associated with spermatogenesis. Recent research using seminal plasma from TESE procedures has identified a panel of lncRNAs that could predict spermatozoa recovery more accurately than sex hormone levels, offering a new avenue for pre-surgical evaluation in NOA patients (64).

CircRNAs have a role in spermatogenesis and are detectable in seminal plasma. They are resistant to degradation by RNA exonucleases, making them stable markers. A recent study identified three circRNAs with the potential to predict TESE outcomes, although their functions and connections to spermatogenesis require further investigation (65).

Finally, tsRNAs, emerging as key players in gene regulation, are involved in sperm maturation and are abundant in seminal plasma. A recent study found higher levels of a specific tsRNA (RF-Val-AAC-010) in NOA patients with successful micro-TESE, suggesting its utility as a biomarker for predicting TESE outcome. This tsRNA was also shown to participate in spermatogenesis, strengthening its potential as a predictive tool (66).

Overall, these RNA molecules in seminal plasma can be a non-invasive way to forecast the success of TESE in NOA patients, thus significantly impacting on clinical decision-making and patient counseling. However, due to the variability and preliminary nature of current studies, larger and more comprehensive investigations are needed to establish their clinical utility.

Limitations: the application of transcriptomics for predicting sperm retrieval in clinical settings faces several limitations:

-Moderate specificity and sensitivity of predictive models: current models based on transcriptomic data, including serum hormones, show only moderate specificity and sensitivity for predicting the presence of testicular spermatozoa in NOA patients (**Table 1**). This suggests that while transcriptomics provides valuable insights, its predictive power in clinical practice is not yet optimal.
-Complexity of seminal plasma transcriptome: the seminal plasma transcriptome in NOA patients is complex, encompassing a wide array of RNAs involved in various regulatory functions. Despite ongoing research, understanding the exact role and significance of these RNAs in spermatogenesis and their potential use as biomarkers for successful sperm retrieval is challenging.
-Variability in miRNA expression: several studies have shown that miRNAs play a significant role in germ cell development and spermatogenesis. However, the expression of miRNAs can be highly variable among NOA patients. While some studies have identified potential miRNAs that could serve as biomarkers for successful sperm retrieval, these findings need validation in larger cohorts.
-Lack of large-scale validated studies and methods: many of the transcriptomic studies in NOA are limited by small sample size. Larger-scale studies are required to validate the potential use of biomarkers and improve their applicability in clinical settings. Variability can also be based on the technical limitations of different applied methods (Real-time PCR, RNASeq, Nanostring technologies).

These limitations indicate that while transcriptomic approaches hold promise for improving sperm retrieval predictions in NOA patients, more research and development are needed to enhance their clinical utility.

### 3.4 Proteomics

Over the past two decades, there have been numerous proteomics studies investigating potential biomarkers for azoospermia. These studies have systematically examined human samples, aiming to identify biomarkers that can provide insights into azoospermia without the need for invasive procedures​​. Proteomic biomarkers include proteins in the seminal plasma that correlate with sperm health and spermatogenesis, and reproductive hormones, like testosterone, FSH, LH, that can be detected in blood and saliva.

Seminal plasma contains proteins in a concentration of 35–55 mg/mL, with a mix of testicular, epididymal, seminal vesicle, and prostate proteins. About 60% of these proteins contribute to its enzymatic activity, including hydrolases and peptidases (67, 68)​​.

Various proteins of testicular and epididymal origin have been identified in seminal plasma, which can help distinguish between OA and NOA​​. Significant proteins that have been studied in this context include:

-L-Lactate Dehydrogenase C (LDHC): found in spermatocytes and spermatids, playing a key role in glycolysis and sperm motility, it is absent in men with OA and NOA, its production is directly proportional to the intensity of spermatogenesis​​ (69).
-Phosphoglycerate kinase 2 (PGK2): a key enzyme in glycolysis, crucial for sperm development and motility, primarily expressed in elongated spermatids​​ (70).
-Transketolase-like protein 1 (TKTL1): its expression begins in spermatogonia and is involved in connecting the pentose cycle with the glycolytic pathway. TKTL1 levels can help distinguish subtypes of NOA​ (71)​. Interestingly, pathogenic variants of the *TKTL1* gene were found in very few azoospermic patients (72).
-Testis expressed 101 (TEX101): a germ cell-specific protein in testes, crucial during sperm capacitation and binding to zona pellucida. The levels of TEX101 in seminal plasma are indicative of spermatogenesis status​​, with high levels suggesting normal spermatogenesis and low levels indicating Sertoli cell-only syndrome or maturation arrest​​ (73).
-Extracellular matrix protein 1 (ECM1): has been identified as a significant biomarker in the diagnosis of NOA. A study involving 119 seminal plasma samples from men with both normal spermatogenesis and azoospermia identified ECM1, along with TEX101, as key in differentiating between OA and NOA (74). They found that at a cutoff level of 2.3 μg/ml of ECM1 could distinguish OA from normal spermatogenesis with 100% specificity and OA from NOA with 73% specificity at 100% sensitivity. This suggests that ECM1 plays a critical role in the molecular processes underlying NOA and has potential clinical significance in diagnosing and differentiating various forms of azoospermia (2, 72).

Limitations:

-Identification and detection challenges: there is a need for identifying as many proteins as possible, which is not feasible for a large number of patients due to time and financial constraints. Moreover, the stability of individual proteins in seminal plasma can vary due to high proteolytic activity, which complicates the reliability of the tests.
-Need for more cost-effective methods: to increase the number of patients tested, more cost-effective methods are necessary. However, such methods may detect fewer protein species, requiring careful selection and filtering of biomarker candidates (**Table 1**).
-Sample size: in proteomic studies for NOA, a small sample size can significantly impact the generalizability of findings. This limitation is especially crucial in proteomics, where quantitative measurement of proteins is key. Compared to identifying pathogenic mutations associated with NOA, where a few instances can be indicative, proteomics often requires a larger sample size for accurate quantification and validation of protein biomarkers. A small sample may not adequately represent the population, leading to challenges in applying the findings more broadly. This distinction is important in understanding the implications of such studies for clinical application.

Overall, while significant progress has been made in proteomic analysis for identifying biomarkers of male sterility in seminal plasma, these challenges highlight the need for continued research and development in this field.

## 4 Integrating non-invasive biomarkers into diagnostic and therapeutic algorithms in NOA

**Figure 1:**
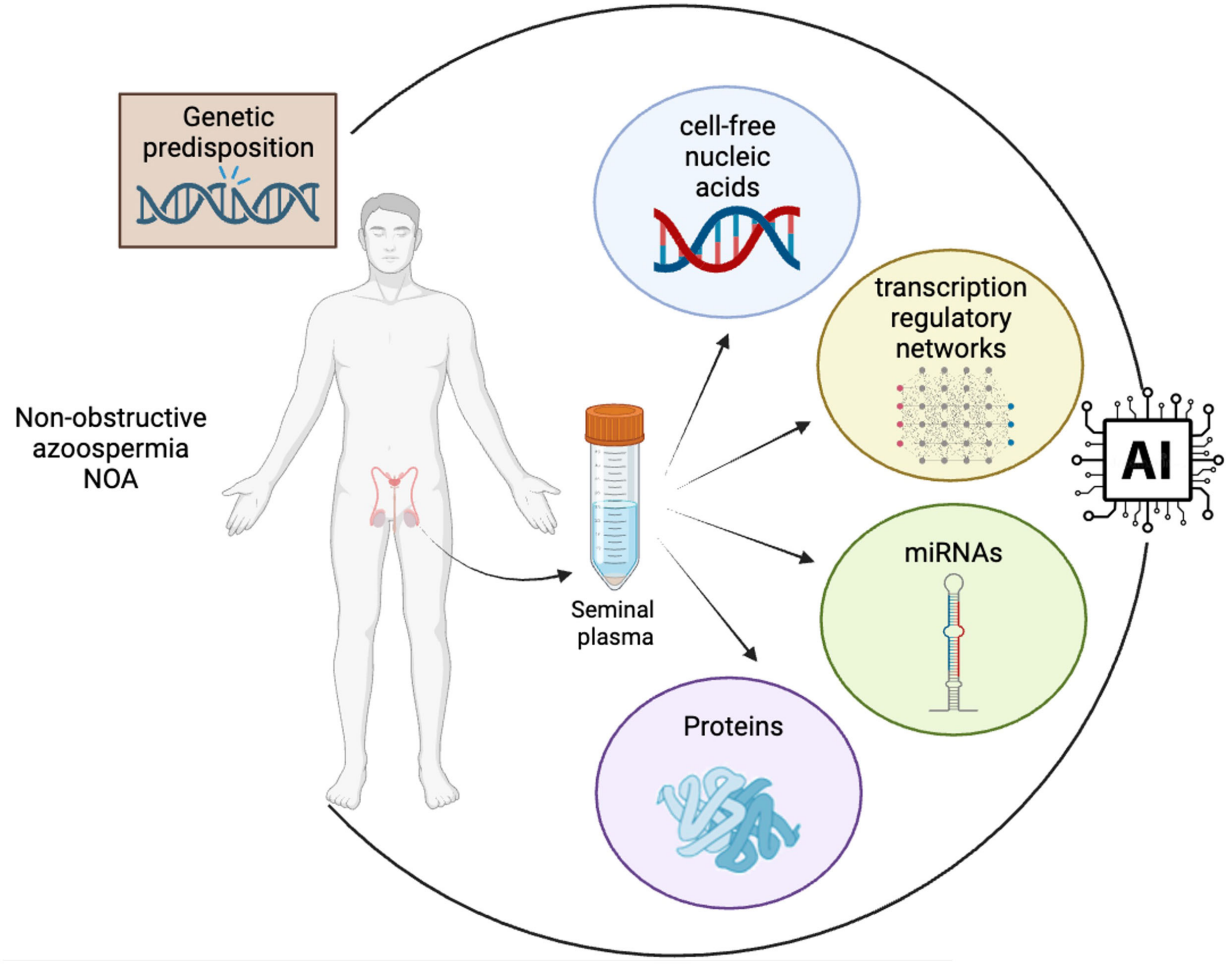
Integration of multidimensional biological data into AI algorithms for advanced biomarker discovery in NOA. The convergence of diverse data types, including clinical data, genetic information and cell-free nucleic acid, proteomic and transcriptomic profiles from seminal plasma, into a central AI system is mandatory for the identification of clinically valuable non-invasive biomarkers in NOA. The flow of data highlights the process of transforming complex biological information into actionable insights through AI-driven analysis, a cornerstone in the field of precision medicine and biomarker research (created in BioRender.com).

In the context of identifying non-invasive biomarkers for sperm retrieval in cases of NOA, the integration of artificial intelligence (AI) with clinical and molecular data presents a transformative approach (**Figure 1**). Some studies demonstrate the application of AI in this field, illustrating how machine learning and bioinformatics can significantly enhance the understanding and diagnosis of NOA (74, 75). The integration of non-invasive biomarkers into the diagnostic and therapeutic algorithms for NOA patients holds the potential to revolutionize the clinical approach to male infertility, and to transform male infertility management into a realm of personalized medicine. By incorporating non-invasive biomarkers into the diagnostic process, clinicians can make more informed decisions about the appropriate treatment strategy for each individual patient, thus enhancing the overall efficiency and success of fertility interventions. By the combined analysis of genetic, proteomic, and metabolomic profiles, clinicians can gain valuable insights into the underlying molecular mechanisms of spermatogenesis specific to each patient.

In the study by Tang et al. (74), five different algorithms using machine learning, including the XG Boost (XGB) algorithm, were employed to identify potential biomarkers for NOA. This approach led to the discovery of a strong correlation of genes such as *IL20RB, C9orf117, HILS1, PAOX*, and *DZIP1* with NOA. Moreover, the study implemented a comprehensive strategy, merging NOA microarray datasets from the GEO database into training sets and a separate validation set. Through differential gene analysis, consensus cluster analysis, and Weighted Gene Co-expression Network Analysis (WGCNA), preliminary signature genes were identified. These genes were then subjected to enrichment analysis for further insights. This methodological approach demonstrates the capacity of AI to integrate and analyze a large amount of clinical and biomarker data, leading to more accurate and efficient identification of potential biomarkers.

Another study (75) aimed to identifying key genes in male infertility, explored DEGs in NOA by microarray datasets from the NCBI’s gene expression omnibus (GEO) database and hub gene analysis. This study highlighted the importance of bioinformatics in unraveling the molecular mechanisms of NOA.

Although these studies collectively represent the forefront of research in this area, demonstrating how AI can be leveraged to enhance our understanding and diagnostic capabilities in the realm of male infertility and reproductive health, limitations of AI in the stratification of NOA patients can be considered. A primary concern is the reliance on datasets that are often limited in size, which can lead to overfitting and reduced generalizability of AI models. Furthermore, the algorithm validation process is crucial yet frequently overlooked, particularly the lack of validation with external datasets, which is essential for ensuring the robustness and applicability of AI tools across different populations and clinical settings. Additionally, the complexity of NOA etiology and the multifactorial nature of male infertility pose challenges for AI models, which may not fully capture the intricacies of the condition without comprehensive, high-quality data. This includes genetic, hormonal, and environmental factors that significantly influence spermatogenesis and patient outcomes. To truly leverage AI in NOA patient care, efforts must be directed towards amassing larger, well-annotated datasets, employing rigorous validation methodologies, and fostering interdisciplinary collaborations to refine AI algorithms. These steps are paramount for advancing AI applications from promising research tools to practical, reliable instruments for clinical decision-making in the management of NOA.

## 5 Roadmap for future development and implementation of non-invasive biomarkers in NOA

Integrating non-invasive biomarkers into the framework of sperm retrieval for NOA patients demands a comprehensive strategy. The first step involves extensive research to confirm the predictive value and clinical effectiveness of these biomarkers in different patient groups. It is crucial to perform large-scale, forward-looking studies to determine the association of these biomarkers in assessing the presence, quality, and quantity of sperm. Equally important is the creation of uniform protocols for evaluating biomarkers, as well as establishing standard reference values, to ensure uniformity and comparability in different lab settings.

Alongside these validation efforts, technological progress is fundamental for the practical application of these biomarkers in clinical environments. Easy-to-use diagnostic tools that quickly and precisely evaluate biomarker profiles will simplify the diagnostic process, ultimately improving patient care. A collaborative effort involving reproductive endocrinologists, geneticists, molecular biologists, bioinformaticians, and imaging experts is vital for a comprehensive approach to biomarker research and validation.

Addressing the regulatory and ethical aspects of using non-invasive biomarkers is also critical. It’s important to establish clear guidelines and procedures for the use of these biomarkers, including considerations for data privacy and patient consent, to ensure their responsible and ethical integration into clinical practices (76).

In summary, incorporating non-invasive biomarkers into the diagnostic and treatment plans for NOA patients is highly promising. With thorough research, technological development, and ethical oversight, these biomarkers could significantly transform sperm retrieval strategies, leading to more individualized, informed, and patient-centric approaches in managing male infertility.

## 6 Conclusions

In this review, we delve into the rapidly evolving domain of non-invasive biomarkers for sperm retrieval in patients with NOA, accentuating the myriad of molecular, genetic, and proteomic factors. A crucial aspect to underscore is the need for thorough genetic diagnostic routines in NOA patients, which are currently underestimating the genetic basis of the condition. Despite the heterogeneous origins of NOA, extensive WES is essential for potentially pinpointing causative genetic variants linked to spermatogenesis. The current standard genetic tests for NOA have a detection rate that fails to detect the presence of additional causative genetic anomalies.

This review also discusses the central role of artificial intelligence (AI) and machine learning in enhancing our comprehension and utilization of these peripheral biomarkers and genetic findings. AI’s ability to analyze complex, multi-omics data sets is crucial in revealing complex biological interactions and pathways involved in NOA. Integrating AI techniques, like machine learning algorithms, to interpret genetic, RNA, and protein data has not only deepened our understanding of NOA’s pathophysiology but also streamlined the identification of potential biomarkers.

This merging role of AI marks a significant leap towards personalized medicine in male infertility. The use of AI allows clinicians and researchers to navigate the complex NOA landscape with more accuracy, potentially leading to more tailored and effective treatment approaches. As AI technology continues to evolve and integrates into clinical practice, it will refine our methods for diagnosing and treating NOA. Future research should focus not only on broadening the array of non-invasive biomarkers but also on exploiting AI and machine learning to maximize their capabilities. These concerted efforts hold the promise of a more nuanced and patient-centered approach to NOA management, heralding significant progress in reproductive medicine.

## Author contributions

LF: Data curation, Writing – original draft. SS: Writing – original draft, Validation. CP: Writing – review & editing. GC: Conceptualization, Data curation, Supervision, Validation, Writing – original draft. MM: Conceptualization, Data curation, Supervision, Validation, Writing – original draft.

## References

[1] Agarwal A Baskaran S Parekh N Cho CL Henkel R Vij S. Male infertility. Lancet. (2021) 397:319–33. doi: 10.1016/S0140-6736(20)32667-2 33308486

[2] Andrade DL Viana MC Esteves SC. Differential diagnosis of azoospermia in men with infertility. J Clin Med. (2021) 10:3144. doi: 10.3390/jcm10143144 34300309 PMC8304267

[3] Esteves SC. Clinical management of infertile men with nonobstructive azoospermia. Asian J Androl. (2015) 17:459–70. doi: 10.4103/1008-682X.148719 PMC4430952 25677138

[4] Caroppo E Colpi GM. Update on the management of non-obstructive Azoospermia: current evidence and unmet needs. J Clin Med. (2021) 11:62. doi: 10.3390/jcm11010062 35011799 PMC8745473

[5] Matzuk MM Lamb DJ. The biology of infertility: research advances and clinical challenges. Nat Med. (2008) 14:1197–213. doi: 10.1038/nm.f.1895 PMC3786590 18989307

[6] Minhas S Bettocchi C Boeri L Capogrosso P Carvalho J Cilesiz NC. European association of urology guidelines on male sexual and reproductive health: 2021 update on male infertility. Eur Urol. (2021) 80:603–20. doi: 10.1016/j.eururo.2021.08.014 34511305

[7] Cioppi F Rosta V Krausz C. Genetics of Azoospermia. Int J Mol Sci. (2021) 22:3264. doi: 10.3390/ijms22063264 33806855 PMC8004677

[8] Punjani N Kang C Schlegel PN. Two decades from the introduction of microdissection testicular sperm extraction: how this surgical technique has improved the management of NOA. J Clin Med. (2021) 10:1374. doi: 10.3390/jcm10071374 33805395 PMC8037781

[9] Majzoub A Arafa M Clemens H Imperial J Leisegang K Khalafalla K. A systemic review and meta-analysis exploring the predictors of sperm retrieval in patients with non-obstructive azoospermia and chromosomal abnormalities. Andrologia. (2022) 54:e14303. doi: 10.1111/and.14303 34729809

[10] Tang D Li K He X Zhang Y Cao Y. Non-invasive molecular biomarkers for predicting outcomes of micro-TESE in patients with idiopathic non-obstructive azoospermia. Expert Rev Mol Med. (2022) 24:e22. doi: 10.1017/erm.2022.17 35659383

[11] Niederberger C. An Introduction to Male Reproductive Medicine. Cambridge: Cambridge University Press (2011). Available at: https://www.cambridge.org/core/books/an-introduction-to-male-reproductive-medicine/D1566CC656D744AEB1C6EC223FD292C5. doi: 10.1017/CBO9780511736254

[12] Zarezadeh R Fattahi A Nikanfar S Oghbaei H Ahmadi Y Rastgar Rezaei Y. Hormonal markers as noninvasive predictors of sperm retrieval in non-obstructive azoospermia. J Assist Reprod Genet. (2021) 38:2049–59. doi: 10.1007/s10815-021-02176-3 PMC8417206 33791895

[13] Quarantani G Sorgente A Alfano M Pipitone GB Boeri L Pozzi E. Whole exome data prioritization unveils the hidden weight of Mendelian causes of male infertility. A report from the first Italian cohort. PloS One. (2023) 18:e0288336. doi: 10.1371/journal.pone.0288336 37540677 PMC10403130

[14] Kherraf ZE Cazin C Bouker A Fourati Ben Mustapha S Hennebicq S Septier A. Whole-exome sequencing improves the diagnosis and care of men with non-obstructive azoospermia. Am J Hum Genet. (2022) 109:508–17. doi: 10.1016/j.ajhg.2022.01.011 PMC8948161 35172124

[15] Zarezadeh R Nikanfar S Oghbaei H Rastgar Rezaei Y Jafari-Gharabaghlou D Ahmadi Y. Omics in seminal plasma: an effective strategy for predicting sperm retrieval outcome in non-obstructive Azoospermia. Mol Diagn Ther. (2021) 25:315–25. doi: 10.1007/s40291-021-00524-8 33860468

[16] Li HM Wan XY Zhao JY Liang XM Dai Y Li HG. Promising novel biomarkers and therapy targets: The application of cell-free seminal nucleotides in male reproduction research. Transl Res. (2023) 256:73–86. doi: 10.1016/j.trsl.2022.12.006 36586533

[17] Li J Yang F Dong L Chang D Yu X. Seminal plasma biomarkers for predicting successful sperm retrieval in patients with nonobstructive azoospermia: a narrative review of human studies. Basic Clin Androl. (2023) 33:9. doi: 10.1186/s12610-023-00184-0 37076787 PMC10116801

[18] McLachlan RI Rajpert-De Meyts E Hoei-Hansen CE de Kretser DM Skakkebaek NE. Histological evaluation of the human testis–approaches to optimizing the clinical value of the assessment: mini review. Hum Reprod. (2007) 22:2–16. doi: 10.1093/humrep/del279 16887924

[19] Caroppo E Colpi EM Gazzano G Vaccalluzzo L Scroppo FI D’Amato G. Testicular histology may predict the successful sperm retrieval in patients with non-obstructive azoospermia undergoing conventional TESE: a diagnostic accuracy study. J Assist Reprod Genet. (2017) 34:149–54. doi: 10.1007/s10815-016-0812-3 PMC5330973 27655389

[20] Zeadna A Khateeb N Rokach L Lior Y Har-Vardi I Harlev A. Prediction of sperm extraction in non-obstructive azoospermia patients: a machine-learning perspective. Hum Reprod. (2020) 35:1505–14. doi: 10.1093/humrep/deaa109 32538428

[21] Esteves SC Agarwal A. Novel concepts in male infertility. Int Braz J Urol. (2011) 37:5–15. doi: 10.1590/S1677-55382011000100002 21385475

[22] Lotti F Maggi M. Ultrasound of the male genital tract in relation to male reproductive health. Hum Reprod Update. (2015) 21:56–83. doi: 10.1093/humupd/dmu042 25038770

[23] Krausz C Riera-Escamilla A. Genetics of male infertility. Nat Rev Urol. (2018) 15:369–84. doi: 10.1038/s41585-018-0003-3 29622783

[24] Malamud D. Saliva as a diagnostic fluid. Dent Clin North Am. (2011) 55:159–78. doi: 10.1016/j.cden.2010.08.004 PMC3011946 21094724

[25] Krausz C Cioppi F. Genetic factors of non-obstructive azoospermia: consequences on patients’ and offspring health. J Clin Med. (2021) 10:4009. doi: 10.3390/jcm10174009 34501457 PMC8432470

[26] Zitzmann M Aksglaede L Corona G Isidori AM Juul A T’Sjoen G. European academy of andrology guidelines on Klinefelter Syndrome Endorsing Organization: European Society of Endocrinology. Andrology. (2021) 9:145–67. doi: 10.1111/andr.12909 32959490

[27] Kuroda S Usui K Sanjo H Takeshima T Kawahara T Uemura H. Genetic disorders and male infertility. Reprod Med Biol. (2020) 19:314–22. doi: 10.1002/rmb2.12336 PMC7542010 33071633

[28] Vorona E Zitzmann M Gromoll J Schüring AN Nieschlag E. Clinical, endocrinological, and epigenetic features of the 46,XX male syndrome, compared with 47,XXY Klinefelter patients. J Clin Endocrinol Metab. (2007) 92:3458–65. doi: 10.1210/jc.2007-0447 17579198

[29] Xu Y Pang Q. Repetitive DNA sequences in the human Y chromosome and male infertility. Front Cell Dev Biol. (2022) 10:831338. doi: 10.3389/fcell.2022.831338 35912115 PMC9326358

[30] Kang C Punjani N Schlegel PN. Reproductive chances of men with Azoospermia due to spermatogenic dysfunction. J Clin Med. (2021) 10:1400. doi: 10.3390/jcm10071400 33807489 PMC8036343

[31] Salonia A Bettocchi C Capogrosso P Carvalho J Corona G Hatzichristodoulou G. EAU guidelines on sexual and reproductive health. Eur Assoc Urol. (2023).

[32] Krausz C Hoefsloot L Simoni M Tüttelmann F. European Academy of Andrology, European Molecular Genetics Quality Network. EAA/EMQN best practice guidelines for molecular diagnosis of Y-chromosomal microdeletions: state-of-the-art 2013. Andrology. (2014) 2:5–19. doi: 10.1111/j.2047-2927.2013.00173.x 24357628 PMC4065365

[33] Krausz C Chianese C Giachini C Guarducci E Laface I Forti G. The Y chromosome-linked copy number variations and male fertility. J Endocrinol Invest. (2011) 34:376–82. doi: 10.1007/BF03347463 21422806

[34] Houston BJ Riera-Escamilla A Wyrwoll MJ Salas-Huetos A Xavier MJ Nagirnaja L. A systematic review of the validated monogenic causes of human male infertility: 2020 update and a discussion of emerging gene-disease relationships. Hum Reprod Update. (2021) 28:15–29. doi: 10.1093/humupd/dmab030 34498060 PMC8730311

[35] Wang PJ McCarrey JR Yang F Page DC. An abundance of X-linked genes expressed in spermatogonia. Nat Genet. (2001) 27:422–6. doi: 10.1038/86927 11279525

[36] GTEx Portal . Available at: https://www.gtexportal.org/home/.

[37] Ensembl genome browser 110. Available at: https://www.ensembl.org/index.html.

[38] Home - OMIM. Available at: https://www.omim.org/.

[39] Bellil H Ghieh F Hermel E Mandon-Pepin B Vialard F. Human testis-expressed (TEX) genes: a review focused on spermatogenesis and male fertility. Basic Clin Androl. (2021) 31:9. doi: 10.1186/s12610-021-00127-7 33882832 PMC8061069

[40] Wyrwoll MJ Köckerling N Vockel M Dicke AK Rotte N Pohl E. Genetic architecture of Azoospermia—Time to advance the standard of care. Eur Urol. (2023) 83:452–62. doi: 10.1016/j.eururo.2022.05.011 35690514

[41] Akbari A Padidar K Salehi N Mashayekhi M Almadani N Sadighi Gilani MA. Rare missense variant in MSH4 associated with primary gonadal failure in both 46, XX and 46, XY individuals. Hum Reproduction. (2021) 36:1134–45. doi: 10.1093/humrep/deaa362 33448284

[42] Nagirnaja L Lopes AM Charng WL Miller B Stakaitis R Golubickaite I. Diverse monogenic subforms of human spermatogenic failure. Nat Commun. (2022) 13:7953. doi: 10.1038/s41467-022-35661-z 36572685 PMC9792524

[43] Wyrwoll MJ Temel ŞG Nagirnaja L Oud MS Lopes AM van der Heijden GW. Bi-allelic mutations in M1AP are a frequent cause of meiotic arrest and severely impaired spermatogenesis leading to male infertility. Am J Hum Genet. (2020) 107:342–51. doi: 10.1016/j.ajhg.2020.06.010 PMC7413853 32673564

[44] Arafat M Har-Vardi I Harlev A Levitas E Zeadna A Abofoul-Azab M. Mutation in TDRD9 causes non-obstructive azoospermia in infertile men. J Med Genet. (2017) 54:633–9. doi: 10.1136/jmedgenet-2017-104514 28536242

[45] Yatsenko AN Georgiadis AP Röpke A Berman AJ Jaffe T Olszewska M. X-linked TEX11 mutations, meiotic arrest, and azoospermia in infertile men. N Engl J Med. (2015) 372:2097–107. doi: 10.1056/NEJMoa1406192 PMC4470617 25970010

[46] Yang C Lin X Ji Z Huang Y Zhang L Luo J. Novel bi-allelic variants in KASH5 are associated with meiotic arrest and non-obstructive azoospermia. Mol Hum Reproduction. (2022) 28:gaac021. doi: 10.1093/molehr/gaac021 35674372

[47] Hashemi Sheikhshabani S Ghafouri-Fard S Hosseini E Omrani MD. A novel homozygote nonsense variant of MSH4 leads to primary ovarian insufficiency and non-obstructive azoospermia. Mol Biol Rep. (2024) 51:68. doi: 10.1007/s11033-023-09000-4 38175272

[48] Gerlevik U Ergoren MC Sezerman OU Temel SG. Structural analysis of M1AP variants associated with severely impaired spermatogenesis causing male infertility. PeerJ. (2022) 10:e12947. doi: 10.7717/peerj.12947 35341049 PMC8944341

[49] Tu C Wang Y Nie H Meng L Wang W Li Y. An M1AP homozygous splice-site mutation associated with severe oligozoospermia in a consanguineous family. Clin Genet. (2020) 97:741–6. doi: 10.1111/cge.13712 32017041

[50] Colombo R Pontoglio A Bini M. Two novel TEX15 mutations in a family with nonobstructive Azoospermia. Gynecologic Obstetric Invest. (2017) 82:283–6. doi: 10.1159/000468934 28355598

[51] Hou X Zeb A Zhou J Zhang H Shi B Muhammad Z. A homozygous KASH5 frameshift mutation causes diminished ovarian reserve, recurrent miscarriage, and non-obstructive azoospermia in humans. Front Endocrinol. (2023) 9(8):e1003645. doi: 10.3389/fendo.2023.1128362/full PMC9971600 36864840

[52] Zuccarello D Ferlin A Cazzadore C Pepe A Garolla A Moretti A. Mutations in dynein genes in patients affected by isolated non-syndromic asthenozoospermia. Hum Reprod. (2008) 23:1957–62. doi: 10.1093/humrep/den193 18492703

[53] Wang X Li Z Ren Y Jiang T Wang YQ Chen M. The Wilms tumor gene, wt1, is critical for mouse spermatogenesis *via* regulation of sertoli cell polarity and is associated with non-obstructive Azoospermia in humans. PloS Genet. (2013). doi: 10.1371/journal.pgen.1003645 PMC3731222 23935527

[54] Krausz C Riera-Escamilla A Chianese C Moreno-Mendoza D Rajmil O Ruiz-Castane E. Whole exome sequencing in non-obstructive azoospermia allows the identification of a high-risk subgroup of infertile men for undiagnosed Fanconi Anemia, a cancer-prone disease. Endocrine Abstracts. (2018). doi: 10.1530/endoabs.56.OC2.3

[55] Gandini L Lombardo F Paoli D Caponecchia L Familiari G Verlengia C. Study of apoptotic DNA fragmentation in human spermatozoa. Hum Reproduction. (2000) 15:830–9. doi: 10.1093/humrep/15.4.830 10739828

[56] Stroun M Lyautey J Lederrey C Olson-Sand A Anker P. About the possible origin and mechanism of circulating DNA: Apoptosis and active DNA release. Clinica Chimica Acta. (2001) 313:139–42. doi: 10.1016/S0009-8981(01)00665-9 11694251

[57] Kist M Vucic D. Cell death pathways: intricate connections and disease implications. EMBO J. (2021) 40:e106700. doi: 10.15252/embj.2020106700 33439509 PMC7917554

[58] Cortés-Gutiérrez EI de la Vega CG Bartolomé-Nebreda J Gosálvez J. Characterization of DNA cleavage produced by seminal plasma using leukocytes as a cell target. Syst Biol Reprod Med. (2019) 65:420–9. doi: 10.1080/19396368.2019.1645236 31539284

[59] Kumar N Singh NK. Emerging role of novel seminal plasma bio-markers in male infertility: A review. Eur J Obstetrics Gynecology Reprod Biol. (2020) 253:170–9. doi: 10.1016/j.ejogrb.2020.08.015 32871440

[60] Di Pizio P Celton N Menoud PA Belloc S Cohen Bacrie M Belhadri-Mansouri N. Seminal cell-free DNA and sperm characteristic’s: An added biomarker for male infertility investigation. Andrologia. (2021) 53:e13822. doi: 10.1111/and.13822 33040391

[61] Goss DM Vasilescu SA Sacks G Gardner DK Warkiani ME. Microfluidics facilitating the use of small extracellular vesicles in innovative approaches to male infertility. Nat Rev Urol. (2023) 20:66–95. doi: 10.1038/s41585-022-00660-8 36348030

[62] Jia H Wang W Zhou Z Chen Z Lan Z Bo H. Single-cell RNA sequencing technology in human spermatogenesis: Progresses and perspectives. Mol Cell Biochem. (2023) 2. doi: 10.1007/s11010-023-04840-x 37659974

[63] Oluwayiose OA Houle E Whitcomb BW Suvorov A Rahil T Sites CK. Altered non-coding RNA profiles of seminal plasma extracellular vesicles of men with poor semen quality undergoing in *vitro* fertilization treatment. Andrology. (2023) 11:677–86. doi: 10.1111/andr.13295 PMC10017372 36111950

[64] Bonaparte E Moretti M Colpi GM Nerva F Contalbi G Vaccalluzzo L. ESX1 gene expression as a robust marker of residual spermatogenesis in azoospermic men. Hum Reprod. (2010) 25:1398–403. doi: 10.1093/humrep/deq074 20356899

[65] Pansa A Sirchia SM Melis S Giacchetta D Castiglioni M Colapietro P. ESX1 mRNA expression in seminal fluid is an indicator of residual spermatogenesis in non-obstructive azoospermic men. Hum Reproduction. (2014) 29:2620–7. doi: 10.1093/humrep/deu261 25316452

[66] Tang Xj Xiao Qh Wang Xl He Y Tian Yn Xia Bt. Single-cell transcriptomics-based study of transcriptional regulatory features in the non-obstructive azoospermia testis. Front Genet. (2022) 13:875762. doi: 10.3389/fgene.2022.875762 35669193 PMC9163961

[67] Singer R Landau B Joshua H Zukerman Z Pick I Sigienriech E. Protein content of human seminal plasma and spermatozoa in relation to sperm counts. Acta Eur Fertil. (1976) 7:281–4. 1030920

[68] Ješeta M Pospíšilová A Mekiňová L Franzová K Ventruba P Lousová E. Non-invasive diagnostics of male spermatogenesis from seminal plasma: seminal proteins. Diagnostics. (2023) 13:2468. doi: 10.3390/diagnostics13152468 37568830 PMC10417070

[69] Hamil KG Sivashanmugam P Richardson RT Grossman G Ruben SM Mohler JL. HE2beta and HE2gamma, new members of an epididymis-specific family of androgen-regulated proteins in the human. Endocrinology. (2000) 141:1245–53. doi: 10.1210/en.141.3.1245 10698202

[70] Ghanami Gashti N Sadighi Gilani MA Jabari A Qasemi M Feizollahi N Abbasi M. The germ cell-specific markers ZPBP2 and PGK2 in testicular biopsies can predict the presence as well as the quality of sperm in non-obstructive Azoospermia patients. Reprod Sci. (2021) 28:1466–75. doi: 10.1007/s43032-020-00427-9 33507524

[71] Davalieva K Rusevski A Velkov M Noveski P Kubelka-Sabit K Filipovski V. Comparative proteomics analysis of human FFPE testicular tissues reveals new candidate biomarkers for distinction among azoospermia types and subtypes. J Proteomics. (2022) 267:104686. doi: 10.1016/j.jprot.2022.104686 35914715

[72] Malcher A Stokowy T Berman A Olszewska M Jedrzejczak P Sielski D. Whole-genome sequencing identifies new candidate genes for nonobstructive azoospermia. Andrology. (2022) 10:1605–24. doi: 10.1111/andr.13269 PMC9826517 36017582

[73] Korbakis D Schiza C Brinc D Soosaipillai A Karakosta TD Légaré C. Preclinical evaluation of a TEX101 protein ELISA test for the differential diagnosis of male infertility. BMC Med. (2017) 15:60. doi: 10.1186/s12916-017-0817-5 28330469 PMC5363040

[74] Tang Q Su Q Wei L Wang K Jiang T. Identifying potential biomarkers for non-obstructive azoospermia using WGCNA and machine learning algorithms. Front Endocrinol (Lausanne). (2023) 14:1108616. doi: 10.3389/fendo.2023.1108616 37854191 PMC10579891

[75] Alagundagi DB Ghate SD Shetty P Gollapalli P Shetty P Patil P. Integrated molecular-network analysis reveals infertility-associated key genes and transcription factors in the non-obstructive azoospermia. Eur J Obstetrics Gynecology Reprod Biol. (2023) 288:183–90. doi: 10.1016/j.ejogrb.2023.07.023 37549510

[76] Gong D Liu YL Zheng Z Tian YF Li Z. An overview on ethical issues about sperm donation. Asian J Androl. (2009) 11:645–52. doi: 10.1038/aja.2009.61 PMC3735320 19767762

